# Microstructure Refinement of a Transformation-Induced Plasticity High-Entropy Alloy

**DOI:** 10.3390/ma14051196

**Published:** 2021-03-04

**Authors:** Hailong Yi, Daixiu Wei, Renyi Xie, Yifan Zhang, Hidemi Kato

**Affiliations:** 1 State Key Laboratory of Rolling and Automation, Northeastern University, Shenyang 110819, China; yihl@ral.neu.edu.cn (H.Y.); xierenyi0@163.com (R.X.); zhangyifan5252@163.com (Y.Z.); 2 Institute for Materials Research, Tohoku University, 2-1-1 Katahira, Sendai 980-8577, Miyagi, Japan; hikato@imr.tohoku.ac.jp

**Keywords:** high-entropy alloy, hot deformation, dynamic recrystallization, constitutive equation, microstructure

## Abstract

High-entropy alloys (HEAs) have attracted extensive interest due to their unprecedented structure and mechanical performance. We recently proposed a series of novel corich twinning induced plasticity (TWIP) and transformation induced plasticity (TRIP) HEAs with superior tensile properties at room temperature; however, the hot deformation behavior has not been reported. Here, we investigated the dynamic recrystallization behavior and grain refinement of a representative TRIP-HEA, compressed at temperatures of 1123–1273 K with strain rates of 0.1–0.001 s^−1^. We characterized the impact of the temperature and strain rate on the grain structure evolution. A constitutive equation was constructed to reveal the correlations between the flow stress, strain rate, temperature, and strain. The apparent activation energy was estimated to be ~385.7 kJ/mol. The discontinuous dynamic recrystallization played an important role in the grain refinement, particularly at a relatively higher temperature and a lower strain rate, and the volume fraction and morphology of the recrystallized grains exhibited a strong dependency on the Zener–Hollomon parameter. The study provides guidelines for the grain refinement of HEAs through thermomechanical processing.

## 1. Introduction

Metals and alloys are indispensable infrastructural materials for various long-duration load-bearing applications. Conventional alloys are generally designed based on a single host metal combined with minor alloying elements. High-entropy alloys (HEAs) with multiprincipal elements have attracted extensive academic interest after their emergence in the year 2004, and they exhibit unprecedented structures and mechanical properties [1,2]. The equiatomic FeMnCoCrNi Cantor alloy is one of the most interesting HEAs, which has a good face-centered cubic (fcc) phase stability and outstanding mechanical performance even at cryogenic temperatures [2,3,4].

The exceptional combination of tensile strength and ductility is due to the low stacking fault energy (SFE = 25–30 mJ/m^2^), in which dislocation slips followed by mechanical twinning carries the plastic deformation [3,4,5,6]. As is known, the SFE of fcc-metals is a major factor governing the plastic deformation mechanism. The deformation mechanism switches from the dislocation slip to mechanical twinning and then to fcc → hcp (hexagonal close-packed) strain-induced martensitic transformation by gradually lowering the SFE [7,8,9,10]. A high SFE value (>45 mJ/m^2^) results in a perfect dislocation slip, an intermediate SFE value (15–45 mJ/m^2^) leads to the generation of mechanical twinning, and a low SFE value (<15 mJ/m^2^) promotes the fcc → hcp transformation [7]. Therefore, tailoring the SFE is an effective method to optimize the mechanical properties of HEAs.

Recently, we revealed the principles for tuning the SFE of the Cantor HEA, assisted by a combination of thermodynamic calculations, ab initio calculations, and experiments [8,9,10]. The decrease in Mn, Ni, and Fe, as well as the increase in the Co and Cr concentrations reduces the SFE of the system and meanwhile increases the elastic modulus and friction stress for dislocation slip [8,9,10]. Following the guidelines, we designed a series of nonequiatomic corich strong and ductile HEAs. Those HEAs exhibited better room-temperature tensile properties than those of the other fcc-phase HEAs, owing to the twinning-induced plasticity (TWIP) and/or transformation-induced plasticity (TRIP) effect.

The novel-designed high-performance HEAs are convincing candidates for biomedical applications, which could be utilized for fabricating surgical wires and stents, owing to the exceptional plasticity of the alloys. For instance, in a Co_35_Cr_25_Ni_15_Mn_15_Fe_10_ (at.%) TRIP-HEA, the dislocation slip was restricted planarly by the low SFE, and the fcc → hcp transformation proceeded during plastic deformation [9]. However, the yield strength needs to be further improved by reducing the grain size according to the well-known Hall–Petch relationship [11,12]. However, the grain refinement by cold-processing is limited due to the poor workability of the generated brittle hcp-martensites.

On the other hand, thermomechanical processing was often applied in the metal industry for fabricating high-quality products, which can contribute to the dimensional precision concerning the optimal physical and mechanical properties [13,14,15,16]. The understanding of the hot deformation mechanism and dynamic recrystallization (DRX) behavior is a prerequisite for conducting the thermomechanical treatment to optimize the grain size and morphology [14,15,16]. However, the majority of the effort has been devoted to conventional metals and alloys, and only a few studies reported the high-temperature plastic deformation characteristics of HEAs.

Recently, the deformation behavior of the CoCrFeMnNi HEA and the CoCrNi medium entropy alloy at a temperature higher than 1073 K was investigated, and the constitutive relationships between the flow stress, strain, strain rate, and temperature were clarified [17,18,19,20,21,22]. Research reported that the evolution of grain structure is accompanied by the discontinuous dynamic recrystallization (dDRX), in which the newly recrystallized grains were referred to the form at the original grain boundaries (GBs) [17,18,19,20,21,22].

On the other hand, the recrystallized grains did not exhibit a strong texture. The apparent activation energy of the Cantor HEA is approximately 350 mJ/mol [17,18,19,20], which is close to that of the diffusion of Ni in the system [23,24,25]. The steady-state deformation is controlled by the dislocation climb [25]. However, the hot-deformation behavior and microstructure evolutions of the nonequiatomic HEAs, particularly the TRIP-HEA, have not been investigated.

In the present study, we aimed to reveal the hot deformation and DRX behaviors of the representative Co_35_Cr_25_Ni_15_Mn_15_Fe_10_ TRIP-HEA, by compression at a temperature higher than 1123 K with various strain rates. We aimed to construct the constitutive relationships for describing the deformation behaviors and to reveal the microstructure evolution of the HEA. The present study provides guidelines for optimizing the grain morphology and mechanical properties of the TRIP-HEA with thermomechanical processing, such as hot-compression, hot-rolling, and hot-extrusion.

## 2. Materials and Methods

The ingot of the Co_35_Cr_25_Ni_15_Mn_15_Fe_10_ (at.%) TRIP-HEA was fabricated by melting and casting in a high-frequency induction machine, operated under a high-purity argon atmosphere. Then, the ingot with a diameter of ~50 mm was hot-forged to a cross-section of 30 × 30 mm^2^ at 1473 K, followed by solution treatment at 1473 K for 4 h and water quenching to room temperature. Then, cylindrical samples with dimensions of 12 mm (height) × 8 mm (diameter) were sliced from the ingots by electric discharge machining. After that, hot-compression of the cylindrical samples was conducted using a Gleeble 1500 machine (Dynamic systems Inc., Poestenkill, NY, USA).

**Figure 1:**
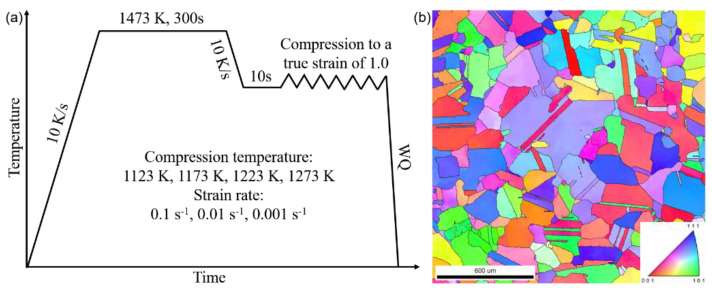
(**a**) Schematic diagram showing the experimental procedure and conditions for the hot compression of the Co_35_Cr_25_Mn_15_Ni_15_Fe_10_ transformation induced plasticity (TRIP) high-entropy alloy (HEA). (**b**) EBSD inverse pole figure (IPF) map shows the initial grain structure before hot compression of the TRIP-HEA.

The procedure of hot compression is described in Figure 1a. All of the samples were heated up to 1473 K with a heating rate of 10 K/s and isothermally held for 300 s at 1473 K to ensure homogeneity. Then, the samples were cooled to compression temperatures of 1123, 1173, 1223, and 1273 with a cooling rate of 10 K/s. Under this routine, the precipitates, as well as the isothermal and athermal martensite, could be eliminated. The compression temperature was determined in the range of 1123–1273 K, based on the following considerations: the dynamic recrystallization occurring at a temperature higher than 1123 K, but rapid grain growth may occur at a temperature higher than 1273 K.

Each sample was held at the temperature for 10 s before compression. Then, the samples were compressed up to a 63% reduction in height (an equivalent true strain of 1.0), with strain rates of 0.1, 0.01, and 0.001 s^−1^. The strain rate was selected to investigate the influence of strain rate on the flow behaviors within the steady-state plastic deformation regime of the HEA. The displacement and load were recorded. The samples were water quenched immediately after the compression. The initial grain structure is shown in Figure 1b, in which the grains are randomly distributed with an average diameter of ~190 μm. In the present study, at least three independent samples were tested for each hot-compression condition to ensure repeatability.

The microstructures of the compressed samples were characterized using a scanning electron microscope (SEM, Hitachi S-3400N, HITACHI, Tokyo, Japan) equipped with an electron backscatter diffraction (EBSD, OIM Analysis, AMETEK Inc., Berwyn, PA, USA) detector. The observation was operated at an acceleration voltage of 20 kV. The cylindrical hot-compressed samples were sectioned vertically through the center parallel to the loading direction, and the central regions were observed. The samples were polished by abrasive paper and mirror-finished using a colloidal silica suspension (OP-U).

## 3. Results

### 3.1. Mechanical Response and Constitutive Relationships

**Figure 2:**
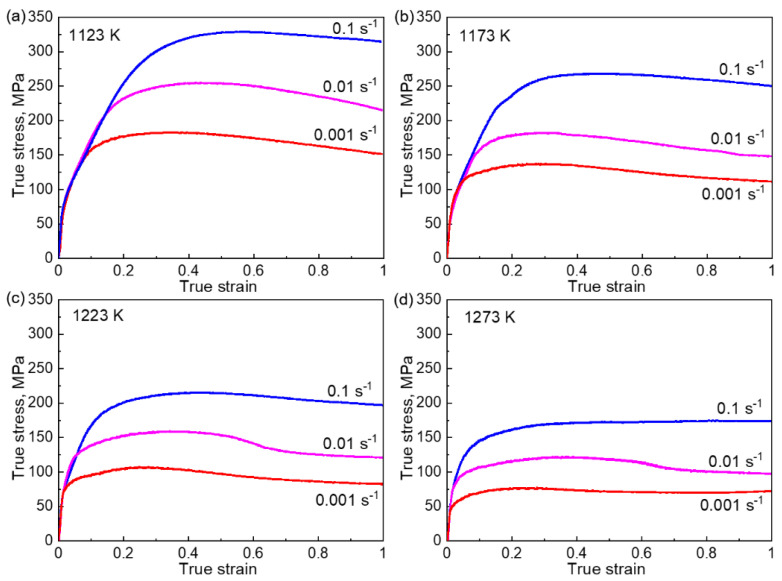
The true stress-strain curves of the Co_35_Cr_25_Mn_15_Ni_15_Fe_10_ TRIP-HEA hot-compressed at (**a**) 1123 K, (**b**) 1173 K, (**c**) 1223 K, and (**d**) 1273 K. The strain rates were 0.1, 0.01, and 0.001 s^−1^.

Figure 2 shows the true stress–strain (*σ-ε*) curves of the Co_35_Cr_25_Ni_15_Mn_15_Fe_10_ (at.%) TRIP-HEA hot-compressed at (a) 1123 K, (b) 1173 K, (c) 1223 K, and (d) 1273 K. The strain rates for the compression were 0.1 s^−1^, 0.01 s^−1^, and 0.001 s^−1^. As is seen from the *σ-ε* curves, the flow stress (*σ*) increased with the increase in the strain (*ε*) at the initial stage of deformation and then reached a peak value. The work hardening was caused by the formation and multiplication of dislocations during the plastic deformation. The early formed dislocations blocked the motion of new dislocations, leading to an increase in flow stress for the further deformation.

**Figure 3:**
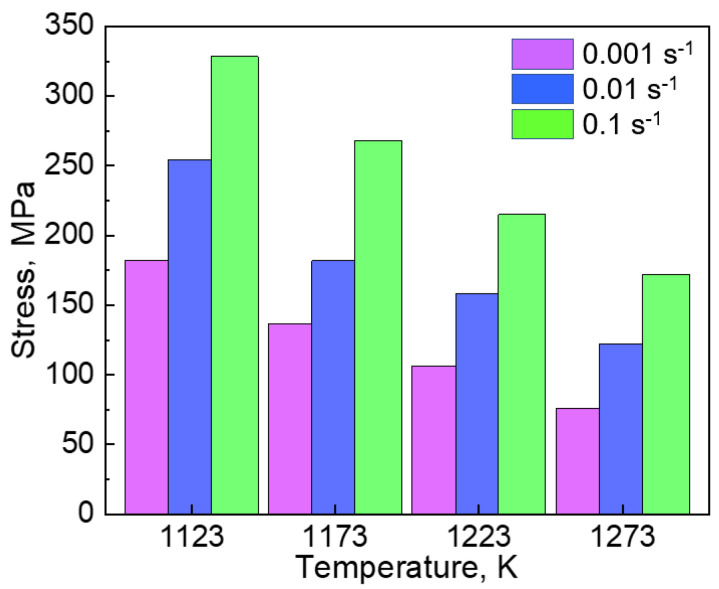
The peak flow stress (*σ*_max_) of the Co_35_Cr_25_Mn_15_Ni_15_Fe_10_ TRIP-HEA hot-compressed at 1123, 1173, 1223, and 1273 K. The strain rates were 0.1, 0.01, and 0.001 s^−1^.

After the peak, the flow stress decreased gradually with the increase in the strain (flow softening), which indicates a dynamic recovery (DRV) of the dislocations or DRX proceeds at this stage. The discontinuous yielding phenomenon reported in hcp metals [14,15,16] was not observed in the present study. The peak stress at each hot-compression condition is compared in Figure 3. The peak flow stress increased with an increase in the strain rate and/or a decrease in the hot-compression temperature.

During the deformation, the accumulation of dislocations and their DRV proceeded concurrently, which are two well-known competitive processes. At a high strain rate or a low temperature, the motion of dislocations is restricted, and only a small number of dislocations was recovered. This contributes to the generation of a high density of dislocations and a large strain hardening rate, leading to a high peak stress. However, the DRV of dislocations was more prevalent at a high temperature or low strain rate, resulting in low peak stresses due to the relatively weak strain hardening.

To reveal the relationship of the flow stress (σ), strain (ε), strain rate ($\dot{\varepsilon }$), and temperature (T) during hot-compression of the TRIP-HEA, a constitutive equation was constructed. Here, we applied the widely accepted Arrhenius-type hyperbolic-sine relationship to reveal the correlation [26]

(1) $$\dot{\varepsilon }=A_{1}{\left[sinh\left(\alpha \sigma \right)\right]}^{n}exp\left(-Q_{def}/RT\right)$$

where the *A*_1_, *α*, and *n* are material constants, *Q_def_* is the apparent activation energy (J·mol^−1^), and *R* is the gas constant (8.314 J·K^−1^mol^−1^).

The correlation of flow stress with the strain rate can also be expressed as follows [27,28]

(2) $$\dot{\varepsilon }=A_{2}\sigma^{n_{1}}\;(\mathrm{low}\;\mathrm{stress})$$

(3) $$\dot{\varepsilon }=A_{3}\exp\left(\beta \sigma \right)\;(\mathrm{high}\;\mathrm{stress})\;$$

where the *A*_2_, *A*_3_, *n*_1_, and *β* are material constants, and *α = β*/*n*_1_.

We used the *σ*-*ε* curves in Figure 2 to determine the material constants of the TRIP-HEA. The value of α was calculated to be 0.00581 by taking the log function on both sides of Equations (2) and (3). Taking the log function on both sides of Equation (1), we obtain

(4) $$n={\langle \partial ln\dot{\varepsilon }/\partial \left(sinh\left(\alpha \sigma \right)\right)\rangle }_{T}$$

(5) $$Q_{def}=nR{\langle \partial \left(sinh\left(\alpha \sigma \right)\right)/\partial \left(1/T\right)\rangle }_{\dot{\varepsilon }}$$

**Figure 4:**
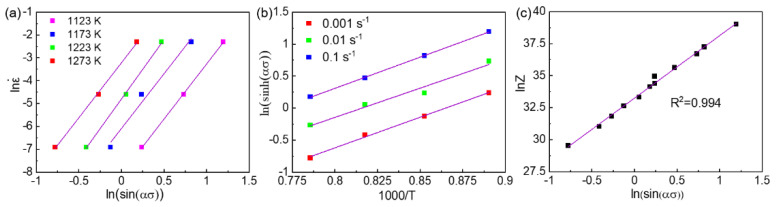
The relationship and linear fitting between (**a**) *ln*$\dot{\varepsilon }$ vs. *ln*[*sin*(ασ)], (**b**) *ln*[*sinh*(ασ)] vs. (1000/*T*), and (**c**) *lnZ* vs. *ln*[*sinh*(ασ)] of the Co_35_Cr_25_Mn_15_Ni_15_Fe_10_ TRIP-HEA. The hot-compression was conducted at 1123, 1173, 1223, and 1273 K with strain rates of 0.1, 0.01, and 0.001 s^−1^, respectively.

To obtain the value of the exponent n, we plotted the relationship of *ln*($\dot{\varepsilon }$) and *lnsinh*(*ασ*) at different temperatures and linear fitted as shown in Figure 4a. The average value of the slopes (*n*) was measured to be 4.884. On the other hand, the correlation of *lnsinh*(*ασ*) with 1000/T at different strain rates was plotted and linearly fitted as shown in Figure 4b. The value of apparent activation energy $Q_{def}$ was measured to be 385.659 kJ/mol.

The strain rate and deformation temperature can be correlated with a simple Zener–Hollomon parameter (*Z*) [29,30],

(6) $$Z=\dot{\varepsilon }exp\left(Q_{def}/RT\right)$$

Comparing Equations (1) and (6) and taking the log function on both sides, we obtain

(7) $$lnZ=lnA_{3}+nln\left(sinh\left(\alpha \sigma \right)\right)$$

The hot-deformation condition can be described by using the *Z* value, in which a large *Z* value represents a high strain rate and/or low deformation temperature, whereas a small *Z* value indicates a low strain rate and/or high temperature. The correlation of *lnsinh(ασ)* with *lnZ* is plotted and fitted in Figure 4c. The coefficient of the fitting was 0.994, indicating a significant linear dependence. Thus, the value of *A*_1_ was 2.7505 × 10^14^.

Therefore, the constitutive relationship of the Co_35_Cr_25_Ni_15_Mn_15_Fe_10_ TRIP-HEA hot-compressed at the temperature of 1123–1273 K with a strain rate of 0.001–0.1 s^−1^ can be expressed as:

(8) $$\dot{\varepsilon }=2.7505\;\times \;10^{14}{\left[sinh\left(0.005816\sigma \right)\right]}^{4.884}exp\left(-385659/RT\right)$$

### 3.2. Microstructure Evolution and DRX

**Figure 5:**
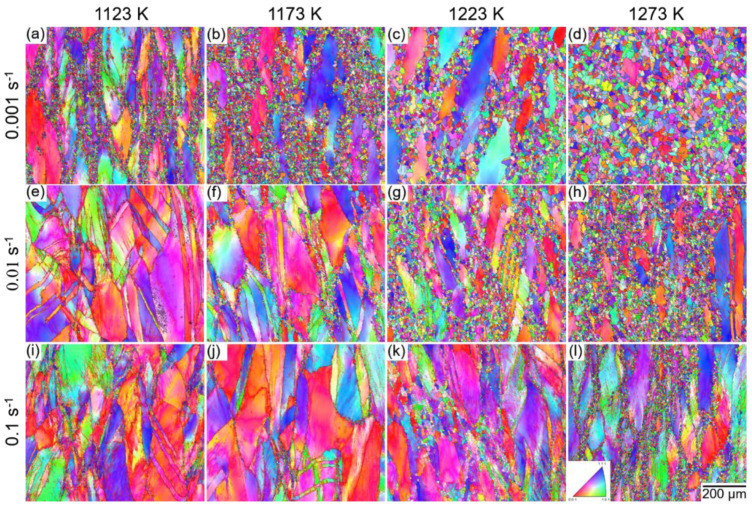
Electron backscatter diffraction (EBSD) inverse pole figure (IPF) maps of the Co_35_Cr_25_Mn_15_Ni_15_Fe_10_ TRIP-HEA hot-compressed at (**a**,**e**,**i**) 1123 K, (**b**,**f**,**j**) 1173 K, (**c**,**g**,**k**) 1223 K, and (**d**,**h**,**l**) 1273 K with strain rates of (**a**–**d**) 0.001 s^−1^, (**e**–**h**) 0.01 s^−1^, and (**i**–**l**) 0.1 s^−1^.

We observed the grain structures of the TRIP-HEA after hot-compression to a true strain of 1.0 by using electron backscatter diffraction (EBSD). Figure 5 shows the EBSD inverse pole figure (IPF) maps of HEA after hot-compression at (a,e,i) 1123 K, (b,f,j) 1173 K, (c,g,k) 1223 K, and (d,h,l) 1273 K with strain rates of (a–d) 0.001 s^−1^, (e–h) 0.01 s^−1^, and (i–l) 0.1 s^−1^. The color in the IPF maps represents the crystallographic orientation of grains. It can be seen that a necklacelike structure was formed especially at a medium *Z* value (a–c,f,g,k,l), which was composed of fine equiaxed grains and coarse elongated grains.

The necklace structure was produced by the formation of DRXed grains at the GBs of the initial coarse grains [31]. The DRX corresponds to the flow softening in the *σ-ε* curves shown in Figure 2. A relatively small amount of DRX grains were formed when the sample was compressed at a large *Z* value (e,i,j), whereas the necklacelike structure disappeared at a very small *Z* value (d,h). We conclude that the progress of DRX was affected by the Z value, in which a medium *Z* value led to the formation of a necklacelike structure (partial DRX), and a small *Z* value resulted in a full recrystallization structure. The DRXed grains did not possess a preferential orientation relationship with the initial coarse grains, i.e., no obvious texture was observed in the samples. Annealing twins were frequently observed in the DRXed grains, which indicates a low SFE of the TRIP-HEA.

**Figure 6:**
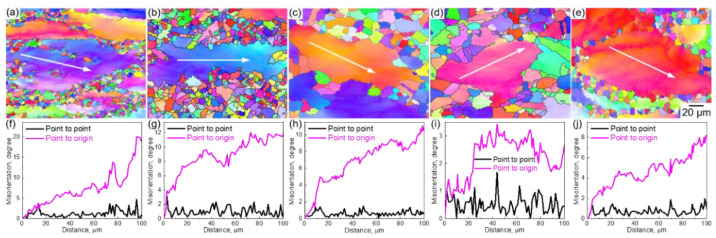
(**a**–**e**) EBSD IPF maps of the Co_35_Cr_25_Mn_15_Ni_15_Fe_10_ TRIP-HEA hot-compressed at (**a**) 1123 K, (**b**) 1173 K, (**c**) 1223 K, (**d**) 1273 K with a strain rate of 0.001 s^−1^ and at (**e**) 1273 K with a strain rate of 0.1 s^−1^. (**f**–**j**) The corresponding point to point and point to origin misorientation profiles along the arrowheads inserted in the coarse grains.

On the other hand, we investigated the characteristics of the unrecrystallized coarse grains in the necklace structure. Figure 6a–e displays the IPF maps of the representative grains of the HEA compressed at (a) 1123 K, (b) 1173 K, (c) 1223 K, and (d) 1273 K with a strain rate of 0.001 s^−1^ and at (e) 1273 K with a strain rate of 0.1 s^−1^. The corresponding misorientation profiles along the arrowheads inserted in the grains are shown in Figure 6f–j, including the point-to-point and point-to-origin misorientations. The coarse grains were surrounded by DRXed grains.

The DRXed grains contained small lattice distortions; however, the coarse grains contained large distortions. The coarse grains were deformed and rotated by the accumulation and rearrangement of dislocations during the hot-compression. By comparing the IPF maps, we found that the misorientation angle tended to decrease with the increase in temperature under the same strain rate (0.001 s^−1^). The misorientation angle tended to increase by increasing the strain rate under the same temperature (1273 K). The deformation of the grains is a competitive process of the dislocation multiplication and DRV, which was affected by the *Z* value (strain rate and temperature).

**Figure 7:**
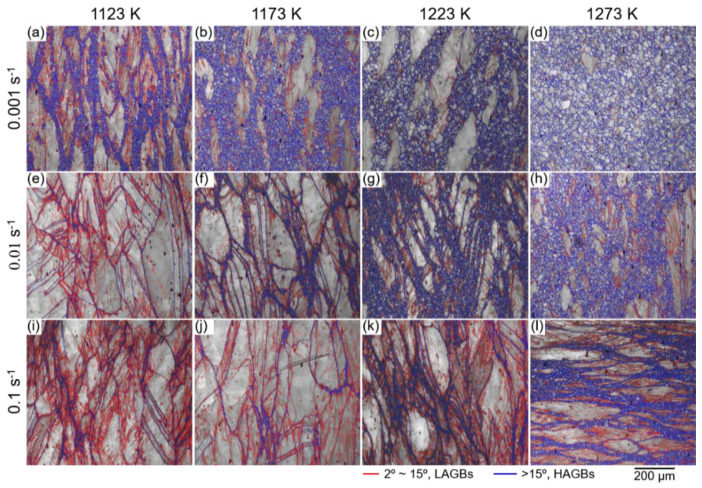
EBSD IQ maps of the Co_35_Cr_25_Mn_15_Ni_15_Fe_10_ TRIP-HEA hot-compressed at (**a**,**e**,**i**) 1123 K, (**b**,**f**,**j**) 1173 K, (**c**,**g**,**k**) 1223 K, and (**d**,**h**,**l**) 1273 K with strain rates of (**a**–**d**) 0.001 s^−1^, (**e**–**h**) 0.01 s^−1^, and (**i**–**l**) 0.1 s^−1^, respectively.

Figure 7 displays the EBSD image quality (IQ) maps of the TRIP-HEA hot-compressed to a true strain of 1.0 at (a,e,i) 1123 K, (b,f,j) 1173 K, (c,g,k) 1223 K, and (d,h,l) 1273 K with strain rates of (a–d) 0.001 s^−1^, (e–h) 0.01 s^−1^, and (i–l) 0.1 s^−1^. In the IQ maps, the blue lines represent high angle grain boundaries (HAGBs), in which the misorientation angle is higher than 15° (θ ≥ 15°). The red lines display the low angle grain boundaries (LAGBs) with misorientation angles 2 ≤ θ < 15°. The necklacelike structure was clearly observed in the samples after hot-compression at a medium *Z* value. The fine grains were mainly surrounded by HAGBs, in which a very small amount of LAGBs was found within the grains.

However, a large number of LAGBs was observed within the unrecrystallized coarse grains. The fines grains were formed by the dDRX; meanwhile, the coarse grains were subdivided into several subgrains surrounded by LAGBs by continuous dynamic recrystallization (cDRX). A larger amount of necklacelike structures was observed in Figure 7a–c,g,l compared with in Figure 7e,f,i,j, indicating that a higher fraction of dDRX proceeded when the HEA was compressed under those conditions. When the HEA was hot-compressed at a large *Z* value, a larger fraction of LAGBs was formed, which means the initial grains were plastically deformed severely under the conditions.

**Figure 8:**
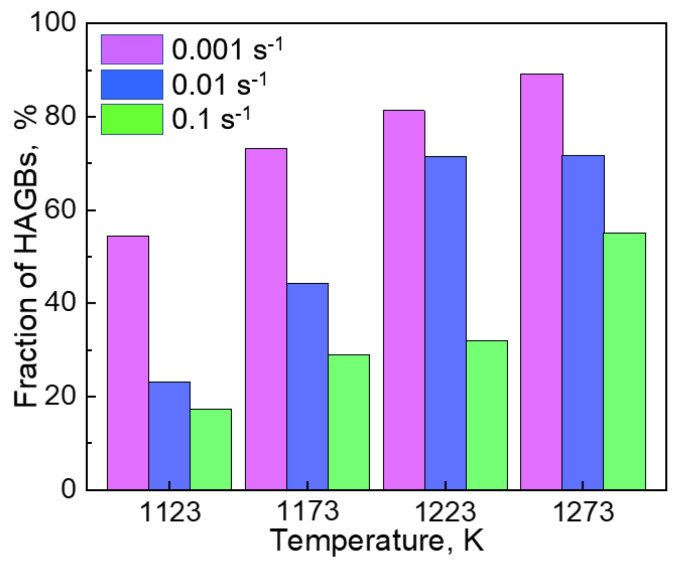
The length fraction of high angle grain boundaries (HAGBs) in the samples hot-compressed at 1123, 1173, 1223, and 1273 K with strain rates of 0.001, 0.01, and 0.1 s^−1^.

Figure 8 displays the fraction of HAGBs in the samples after hot-compression to a true strain of 1.0 at 1123, 1173, 1223, and 1273 K with strain rates of 0.001, 0.01, and 0.1 s^−1^,. At a certain strain rate, the fraction of HAGBs increased with the increase in temperature. On the other hand, the fraction decreased with the increase in the strain rate at a certain temperature. We concluded that a high temperature and a low strain rate were the appropriate conditions for obtaining recrystallized grain structures.

**Figure 9:**
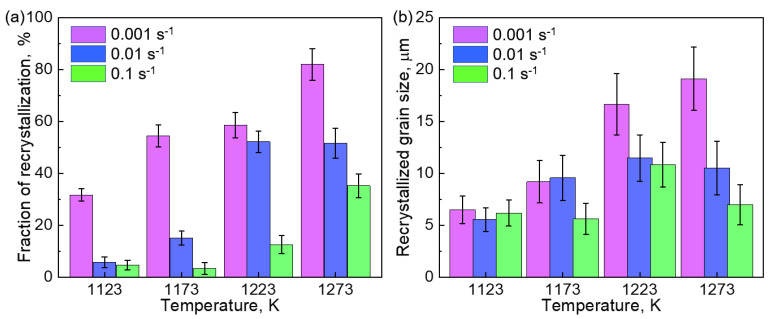
The volume fraction (**a**) and average diameter (**b**) of the DRXed grains in the hot-compressed Co_35_Cr_25_Mn_15_Ni_15_Fe_10_ TRIP-HEA. The compression temperatures were 1123, 1173, 1223, and 1273 K, and the strain rates were 0.001, 0.01, and 0.1 s^−1^.

Figure 9 shows the volume fraction (a) and average diameter (b) of the DRXed grains in the HEA compressed at 1123, 1173, 1223, and 1273 K with strain rates of 0.001, 0.01, and 0.1 s^−1^. The volume fraction of the DRXed grains increased with the increase in temperature but decreased with the increase in the strain rate. For instance, the volume fraction was only 4.8% (smallest) when the HEA was compressed at 1123 K and 0.1 s^−1^, but the value was increased to 82% (largest) when the HEA was compressed at 1273 K with a strain rate of 0.001 s^−1^.

Grain growth often occurs at elevated temperatures to minimize the energy of GBs. As indicated in Figure 9b, the DRXed grains demonstrated preferential growth at a higher temperature and a low strain rate, which resulted in a relatively large grain size. In all the investigated conditions, the average diameter of the DRXed grains ranged from 5 to 20 μm. The comparison demonstrates that a higher fraction of DRX progressed at a relatively high temperature and small strain rate, but the produced DRXed grains became larger due to the grain coarsening.

## 4. Discussion

### 4.1. Mechanical Responses during Hot-Compression

The flow behavior of the Co_35_Cr_25_Ni_15_Mn_15_Fe_10_ TRIP-HEA was investigated by hot-compression, and a constitutive equation was successfully established for describing the correlations between the mechanical response and hot-compression condition. All of the *σ-ε* curves showed work hardening behavior at a small strain, in which the flow stress increased with the increase in strain before reaching a peak value. After the peak stress, the curves showed a flow softening behavior with an increase in strain.

There are several factors affecting the flow behaviors of metals and alloys, including the intrinsic factors of materials, such as the SFE, activation energy, and secondary phase, along with the external factors, such as the temperature, strain rate, and strain [13]. In the present study, the TRIP-HEA was fcc single-phase, and thus the influence of the second phase could be ignored.

The influence of the SFE and activation energy should be taken into consideration. During hot-compression, the accumulation and interaction of dislocations carried the plastic deformation at a small strain [13]. However, the DRV of dislocations always proceeds concurrently at elevated temperatures, which reduces the dislocation density and the stored energy by a mechanism of dislocation climb, cross-slip, and glide [32].

In metals with a high SFE, such as aluminum (160–250 mJ/m^2^), the climb and cross-slip of dislocations occurs easily, and thus the DRV is rapid and extensive at elevated temperatures [13,32,33]. The dislocation density rises at the beginning of deformation, which increases the driving force and the rate of DRV. When a certain strain is reached, the accumulation and DRV of dislocations reach a dynamic equilibrium. The *σ-ε* curves of high SFE metals often exhibit a strain hardening to a plateau (peak stress), followed by a constant or steady-state flow behavior (the flow stress does not change with the increase in strain) [34,35].

The DRX does not occur under those conditions due to the inadequate stored driving force for its incipience. In this case, subgrains are formed by the equilibrium process of hardening and recovery, in which there is a continual formation and dissolution of LAGBs to a constant density of free dislocations within subgrains [36,37,38,39]. However, the DRV of dislocations is impeded significantly in metals with a low or medium SFE, such as copper (70–78 mJ/m^2^) and nickel (90 mJ/m^2^) [33,40]. In those metals, the dislocation density increases rapidly as the hot-deformation continues at a small strain, in which the *σ-ε* curves exhibit a strain hardening stage, and the flow stress is increased to a peak value at a critical strain [13,40,41].

After that, the DRX occurs during the increase in strain, which significantly consumes the stored energy (dislocation density). The DRX primarily proceeds at the pre-existing grain boundaries, which form fine DRXed grains [42,43,44]. The reduction in the dislocation density decreases the flow stress [45]; however, the grain refinement increases the flow stress [11,12]. The flow stress decreases with the increases of strain, leading to the flow softening behavior in the *σ-ε* curves. The SFE of the Co_35_Cr_25_Ni_15_Mn_15_Fe_10_ TRIP-HEA is much lower than that of the Cantor alloy (~25 mJ/m^2^), and thus the DRV process was notably hindered, and the DRX proceeded during hot-compression. The *σ-ε* curves exhibited strain hardening followed by the DRX-induced flow softening as reported in other low SFE metals.

On the other hand, the apparent activation energy for the hot-deformation of the Co_35_Cr_25_Ni_15_Mn_15_Fe_10_ TRIP-HEA was 385.7 kJ/mol, which is slightly higher than that of the Cantor HEA (~350 kJ/mol) [17,18,19,20]. The activation energies of pure aluminum, copper, and nickel are 142, 197, and 285 kJ/mol, respectively [24,46]. The large activation energy of the TRIP-HEA indicates the sluggish diffusion of elements in the multiprincipal system, which reduces the rate of DRV and postpones the incipience of DRX. As a result, the strain hardening effect was increased. In the present study, the peak stress was much higher than that of the pure metals compressed under the same conditions.

The temperature and strain rate also exposed an apparent effect on the flow behaviors during hot-compression. The onset of DRX occurred only when the value of ρ^3^/$\dot{\varepsilon }$ was larger than a critical value, where the ρ is the dislocation density [47,48,49]. The DRX typically originates at HAGBs, in which the bulging of GBs is the prelude. The stored energy of dislocations (the driving force) and mobile GBs (nucleation sites) are the requirements for DRX. At a certain value of $\dot{\varepsilon }$, the mobility of GBs increases notably with the increase in temperature [13].

Thus, a relatively low critical density of dislocations is required for the nucleation of DRXed grains, and the DRX occurs at relatively low flow stress. When the HEA was hot-compressed at a certain temperature, the mobility of GBs is constant. Thus, a higher density of dislocations is required to reach the critical value of the ρ^3^/$\dot{\varepsilon }$, leading to the DRX occurring at a higher flow stress level. On the other hand, a higher strain rate provides a shorter time for the DRV, thus, promoting the strain hardening to a high value of stress. As a result, the flow stress at a large *Z* value (high strain rate and low temperature) was larger than that at a small *Z* value (low strain rate and high temperature).

The plateau in the *σ-ε* curves demonstrated a steady flow behavior of the HEA under these conditions, which was a dynamic balance of strain hardening and the strain-softening caused by DRV, DRX, and grain growth. The plateaus were more easily formed at a relatively high temperature and low strain rate, in which a high degree of recrystallization occurred as shown in Figure 2. The stress drop more easily occurred at a higher temperature and lower strain rate, which represents the completion of the recrystallization [50].

### 4.2. Influence of Hot-Deformation on Microstructures

In the present, the grain size of the Co_35_Cr_25_Ni_15_Mn_15_Fe_10_ TRIP-HEA was refined by hot-compression at a temperature higher than 1123 K. Homogeneous grain structures with a grain size of ~20 μm were formed after hot-compression to a true strain of 1.0 at 1273 K with a strain rate of 0.001 s^−1^. The samples were water quenched to room temperature immediately after hot-deformation, in which the static recrystallization was unlikely to occur. Therefore, the microstructure change was caused by the DRX, which was proceeded by a mechanism of dDRX and cDRX.

The cDRX was achieved by the geometric dynamic recrystallization and progressive lattice rotation mechanisms. In the former, the serrations of HAGBs are generated by the multiplication and interaction of HAGBs [51,52,53]. In the latter mechanism, the subgrains are generated by the strain-induced misorientation gradient at HAGBs of the original grains [42,54]. The majority of the GBs of the newly formed subgrains are LAGBs. On the other hand, the dDRX was generally proceeded by the nucleation of new dislocation-free grains at the GBs of original grains and DRXed grains as well as the HAGBs created during straining, such as deformation bands or mechanical twins. The dDRXed grains were formed by the bulging of GBs by the strain-induced migration. When the grain size between the initial grains and cDRXed grains is large, a necklacelike structure is formed.

**Figure 10:**
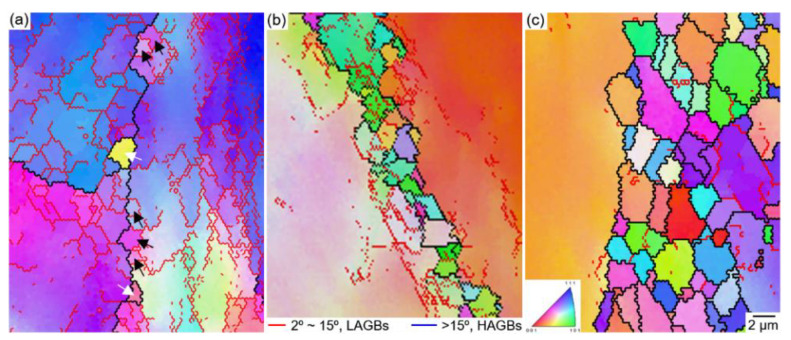
Magnified EBSD IPF maps shows the Co_35_Cr_25_Mn_15_Ni_15_Fe_10_ TRIP-HEA hot-compressed at 1123 K with strain rates of (**a**) 0.1 s^−1^, (**b**) 0.01 s^−1^, and (**c**) 0.001 s^−1^.

Figure 10 shows the magnified EBSD IPF of the TRIP-HEA compressed at 1123 K with strain rates of (a) 0.1 s^−1^, (b) 0.01 s^−1^, and (c) 0.001 s^−1^. As shown in Figure 10a, very fine dDRXed grains (denoted by white arrowheads) with HAGBs were formed at the initial GBs; meanwhile, a large number of cDRXed subgrains (denoted by black arrowheads) with LAGBs were generated at the grain interiors. With the decrease in strain rate, more dDRXed grains with HAGBs were formed at the initial GBs, but the amount of subgrains and LAGBs decreased significantly as indicated in Figure 10b,c.

The necklacelike structure was formed under those conditions. The hot-deformation condition, i.e., the Z-value, affected the obtained microstructures notably. When the HEA was deformed at a large Z-value, a large number of LAGBs were formed as indicated in Figure 7e,i–k and Figure 10a. Thus, the cDRX occurred under those conditions, and subgrains formed in both the regions close to the GBs and in the grain interiors [55]. At a large *Z* value, the DRV and the migration of GBs were impeded due to the low deformation temperature and high strain rate; thus the dDRX was limited, but the cDRX became prevalent.

The cDRX was restrained, but the dDRX was promoted when the HEA was compressed at a small *Z* value. A higher fraction of necklacelike structures and a larger amount of DRXed grains were formed at the GBs of the initial grains as demonstrated in Figure 5, Figure 6, Figure 7, Figure 8, Figure 9 and Figure 10. Due to the high temperature and/or low strain rate, the dislocations were rapidly recovered. The strain-induced continuous lattice rotation became difficult, which prevented the formation of subgrains by the cDRX process.

The good mobility of the initial GBs at a high temperature and/or low strain rate promoted the nucleation of the dDRX. When the HEA was hot-compressed at 1273 K with a strain rate of 0.001 s^−1^, homogeneous grain structures were formed by the dDRX. The size of the dDRXed grains was relatively small, and no obvious grain growth was observed. The grain growth progressed with the diffusion-controlled migration of GBs. In the Cantor alloy, the diffusion coefficient and activation energy of each element were larger than that of the pure fcc-metals and dilute alloys. In the Co_35_Cr_25_Ni_15_Mn_15_Fe_10_ TRIP-HEA, the activation energy for hot-deformation was 385.7 kJ/mol, which is higher than that of the Cantor alloy. Thus, the sluggish diffusion and large activation energy resulted in sluggish grain growth during hot-compression.

## 5. Conclusions

The hot deformation behavior and microstructure evolution of a representative transformation induced plasticity (TRIP) high-entropy alloy (HEA) with a composition of Co_35_Cr_25_Mn_15_Ni_15_Fe_10_ (at.%) were investigated. The hot-compression was conducted to a true strain of 1.0 at 1123, 1273, 1223, and 1473 K. The investigated strain rates were 0.1, 0.01, and 0.001 s^−1^. The main findings are as follows:

1. A constitutive equation that describes the correlation of flow stress with compression condition was successfully acquired. The hyperbolic-sine of the flow stress exhibited a linear correlation with the Zener–Hollomon parameter. The apparent activation energy for hot-deformation was measured to be 385.7 kJ/mol, which is higher than that of the equiatomic CoCrFeMnNi Cantor alloy, demonstrating a sluggish diffusion in the TRIP-HEA.
2. During hot-compression of the TRIP-HEA, the stress–strain curves exhibited a strain hardening at the initial stage followed by a flow softening behavior. The accumulation of dislocations led to strain hardening, whereas the continuous dynamic recrystallization (cDRX) and discontinuous dynamic recrystallization (dDRX) resulted in flow softening. The cDRX preferentially occurred at a large Zener–Hollomon parameter (low temperature and high strain rate), whereas the dDRX became predominant at a small Zener–Hollomon parameter (high temperature and low strain rate).
3. The dynamically recrystallized microstructure was significantly affected by the Zener–Hollomon parameter. A higher fraction of DRXed grains were formed at a smaller Zener–Hollomon parameter. Among the investigated conditions, a temperature of 1273 K and a strain rate of 0.001 s^−1^ were the most appropriate for producing a homogeneous grain structure, in which the grain size was approximately 19 μm after compression to a true strain of 1.0. The DRXed grains exhibited no obvious texture. The results shed light on the modification of grain characteristics through thermomechanical processing.

## Data Availability

All data in this study are available from the corresponding author upon reasonable request.
